# Colonic Gallstone Ileus: Treatment Challenges

**DOI:** 10.7759/cureus.19869

**Published:** 2021-11-24

**Authors:** Teresa Da Cunha, Bashar Sharma, Steven Goldenberg

**Affiliations:** 1 Internal Medicine, University of Connecticut Health, Farmington, USA; 2 Gastroenterology and Hepatology, University of Connecticut Health, Farmington, USA

**Keywords:** cholecystoenteric fistula, gallstone, bowel obstruction, colonic gallstone ileus, gallstone ileus

## Abstract

Intestinal obstruction at the level of the colon is rarely caused by a gallstone. Colonic gallstone is more frequently observed in elderly patients and is associated with high mortality due to treatment challenges. Management with less invasive approaches, including mechanical lithotripsy and endoscopy has been evolving. However, the outcomes are variable, and surgery remains the main cornerstone of treatment. We present a case of an 89-year-old male with gallstone ileus at the level of the sigmoid colon in whom treatment with endoscopy was not successful. We performed an extensive review of the literature to understand the most common presentation, diagnostic modalities, and treatment approach of the sporadic reported cases of colonic gallstone ileus.

## Introduction

Gallstone ileus is a well-characterized pathology in which bowel obstruction occurs secondary to a gallstone impaction. The stone usually travels to the intestine through a cholecystoenteric fistula which typically forms between the gallbladder and the duodenum, but it can form with other sites of the gastrointestinal tract. The most common site of stone impaction is the ileum, however, around 4% can occur in the colon [1]. Elderly patients and especially females are at higher risk for developing colonic gallstone ileus, moreover, they usually present with other comorbidities which contribute to the high morbidity and mortality associated with colonic gallstone ileus. The management of colonic gallstone ileus has been evolving to less invasive approaches with some success with therapeutic colonoscopy, however, surgery remains the cornerstone for its management [2]. We present a case of colonic gallstone ileus and performed an extensive review of the literature to understand the most common presentation, diagnostic modalities, and treatment approach of the sporadic reported cases of colonic gallstone ileus.

## Case presentation

An 89-year-old male with a history of stroke, atrial fibrillation, heart failure, esophageal adenocarcinoma status post radiation, cholecystitis one year prior to presentation (treated with percutaneous cholecystostomy tube that had subsequently been removed), presented to the emergency room with left lower quadrant abdominal pain of two-day duration. He denied nausea, vomiting, diarrhea, constipation, or blood in the stool. He did not have any sick contacts or recent travel. In the emergency room, his temperature was 98.1°F, pulse 80 beats per minute, respiratory rate 16 breaths per minute, and blood pressure 106/67 mmHg. The laboratory blood tests are described in Table 1.

**Table 1:** Blood test results on admission. WBC, white blood cell; BUN, blood urea nitrogen; AST, aspartate aminotransferase; ALT, alanine aminotransferase; ALP, alkaline phosphatase; TBIL, total bilirubin

Blood test	Result	Normal range
WBC (10^9^/L)	8.3	4.5-11
Hemoglobin (g/dL)	11.5	13.5-17.5
Hematocrit (%)	35	41-51
Platelet count (10^3^/mL)	326	150-450
Sodium (mEq/L)	136	135-145
Potassium (mEq/L)	4.8	3.6-5.2
Creatinine (mg/dL)	1.0	0.74-1.35
BUN (mg/dL)	21	6-24
AST (U/L)	20	8-33
ALT (U/L)	9	7-55
ALP (U/L)	176	44-147
TBIL (mg/dL)	0.7	0-1.0

**Figure 1:**
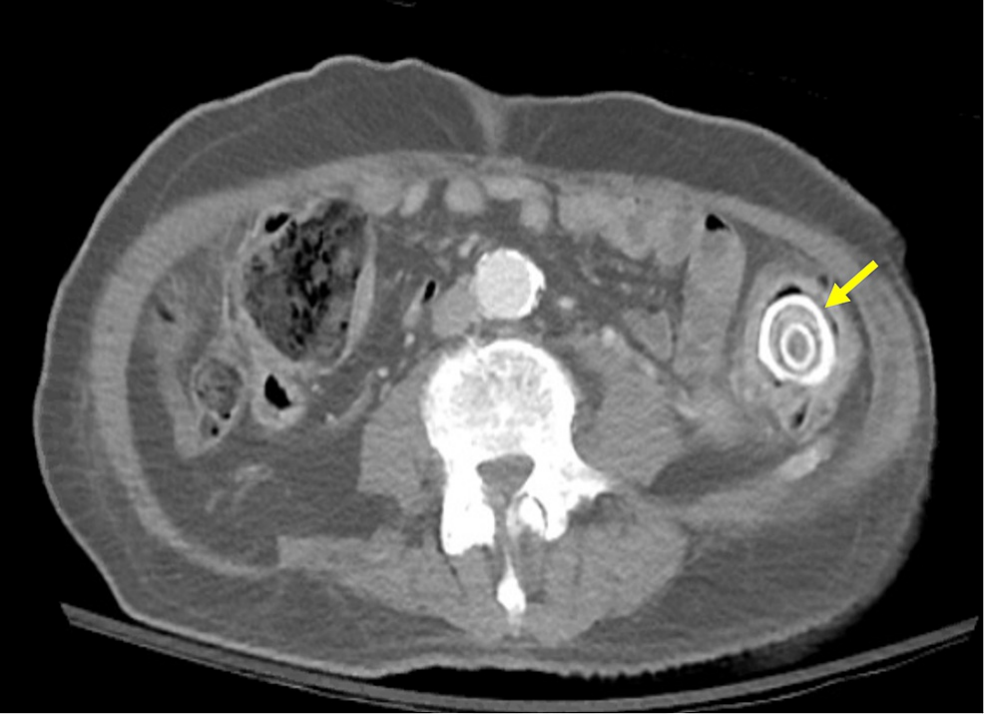
CT abdomen pelvis shows large lamellated stone in the distal descending colon.

**Figure 2:**
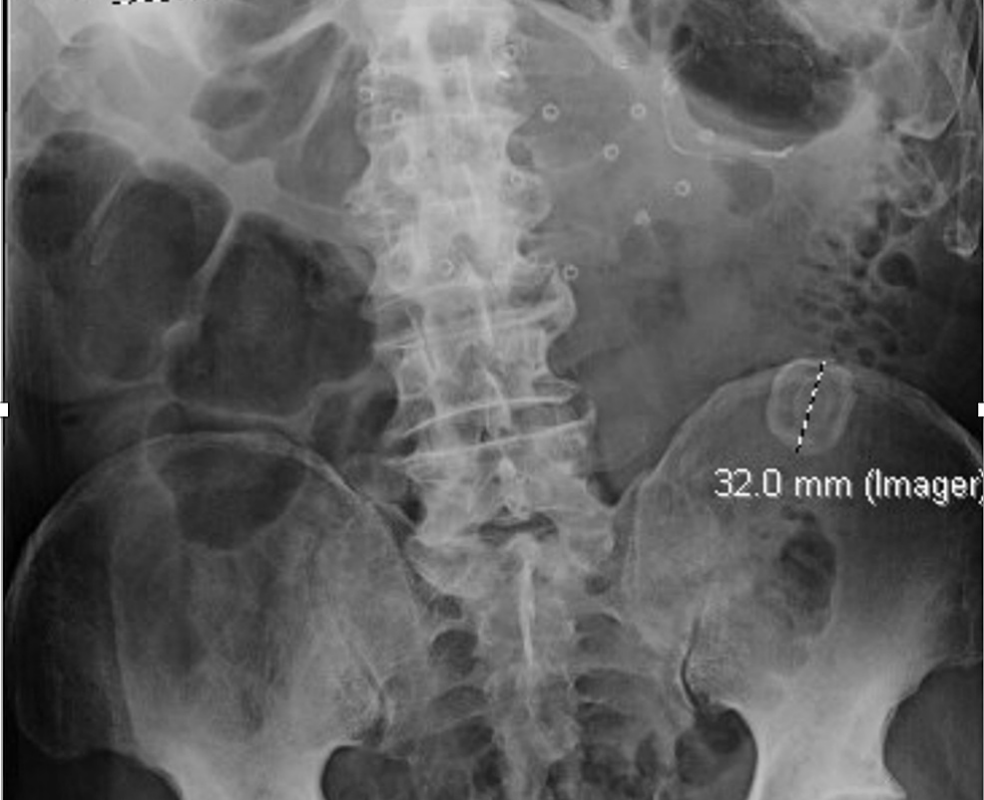
Gastrografin enema showing diverticular structuring and a 32 mm calculus in the descending colon.

A CT of the abdomen and pelvis revealed a fistula between the gallbladder and the large bowel at the hepatic flexure and air in the gallbladder. Marked diverticulosis with wall thickening and fat stranding was seen at the level of the mid to distal descending colon. A large lamellated stone (3 cm x 2.6 cm) was present in the distal descending colon (Figure 1). A gastrografin enema revealed sigmoid colon diverticular stricturing disease (Figure 2).

**Figure 3:**
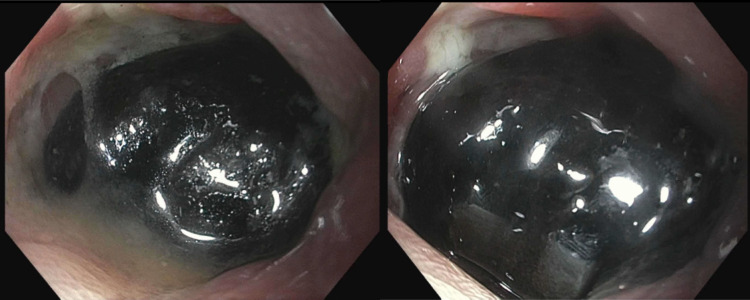
Large pigmented gallstone seen at the sigmoid level during colonoscopy.

Given the patient’s several comorbidities surgery was deferred, the patient was kept nil per mouth and conservative treatment with an aggressive bowel regimen including mineral oil enemas as well as IV hydration was started. However, after four days he did not have any bowel movement and for this reason, total parenteral nutrition was initiated and an endoscopic approach to remove the stone was planned. On the fifth day of hospitalization, a colonoscopy was performed which showed a large black pigmented stone that was completely obstructing the lumen of the sigmoid colon, the surrounding mucosa appeared ulcerated (Figure 3).

Attempts to pass a guidewire proximal to the stone to pursue balloon-assisted dislodgment of the stone or mechanical lithotripsy as well as attempts to capture the stone in a retrieval net were unsuccessful. Consequently, an exploratory laparotomy with partial left colectomy was successfully done, however, the cholecystocolonic fistula was left intact. Later, the pathology results revealed a 3.4 cm x 2.7 cm black stone and a 4.5 cm x 3 cm transmural defect at the colonic wall where the stone was impacted. The patient’s postoperative period was complicated, he did not tolerate oral intake and his nutrition deteriorated substantially despite total parenteral nutrition (TPN). At the request of the patient and the family he opted for inpatient hospice care. Unfortunately, he died 15 days after the surgery.

## Discussion

Gallstone ileus is an extremely rare cause of mechanical bowel obstruction and is responsible for less than 5% of cases of bowel obstruction. However, when stratifying according to age, it is the main cause of intestinal obstruction in up to 25% of patients older than 65 [1]. Moreover, about 0.3%-0.5% of patients with cholelithiasis develop gallstone ileus [3]. Both the study of Rodríguez Hermosa et al. and Clavien et al. reported a higher incidence of this pathology in elderly women with male to female ratios of 5.5:1 and 8.25:1 and a mean age of 76 and 78 years old, respectively [4-5]. These studies, however, lack power owing to its sample size. A recent large study at a national level in the United States showed a lower incidence (0.095%) of gallstone ileus in 3.452.536 cases of mechanical bowel obstruction, from these, more than 70% were elderly women [6]. In the study of Rodríguez Hermosa et al. which reviewed 40 cases of gallstone ileus, around 65% of patients had a history of biliary disease, including cholecystitis, biliary colic, cholelithiasis, or choledocholithiasis. In most cases, a biliary-enteric fistula develops allowing communication between the gallbladder and the intestine and subsequent travel of the gallstones [4]. The most frequent type is a cholecystoduodenal fistula, due to the anatomical proximity [3]. Nonetheless, a cholecystocolonic fistula has been reported and it is the most common association with gallstone impaction in the colon [7].

The location of the gallstone entrapment can occur anywhere from the duodenum to the colon. According to the study of Rodríguez Hermosa et al. the most common location of the gallstone in the gastrointestinal tract was the ileum (62.5%), followed by the jejunum (22.5%), duodenum (7.5%), and colon (2.5%) [4]. However, in the larger study of Reisner et al. that involved 1001 cases of ileus, the ileum also accounted for 60.5% of the cases but a higher percentage of patients (4.1%) had had a colonic gallstone impaction leading to bowel obstruction [1]. The symptomatology of gallstone ileus varies according to the location of the obstruction. Symptoms of intestinal obstruction are commonly reported, others include jaundice and hematemesis. In the case of colonic gallstone ileus, constipation, abdominal pain, and vomiting are frequently reported [7-9].

We performed a review of the literature to further understand the clinical presentation, diagnosis, treatment, and outcomes of patients with gallstone ileus of the colon. EMBASE and PubMed were used to search for articles related to bowel obstruction caused by gallstone impaction in the colon. The following keywords were used: “colonic gallstone ileus,” “gallstone impaction,” “gallstone bowel obstruction,” “sigmoid gallstone ileus,” and "cholecystocolonic fistula." The references of retrieved articles were extensively reviewed for further inclusion of additional cases. A total of 56 cases were found and we also included our case in this review.

The mean age was 80 years old and 74% (n=42) were women. The most commonly reported clinical presentation was abdominal pain (84%), vomiting (67%), and constipation (61%). At least 25% of the patients had a reported history of some type of biliary tract disease including, cholecystitis, symptomatic cholelithiasis, and cholangitis. Elevated inflammatory markers are frequent, from the 22 cases that mentioned the initial blood results, the white blood count ranged from 6.7 x 109/L to 27.4 x 109/L and the average was 14.3 x 109/L. Similarly, from the 16 patients who had a reported C-reactive protein level, 15 had elevated levels which averaged 122 mg/L. 

Given the clinical picture of bowel obstruction, abdominal X-rays are usually performed and the Rigler’s triad (small bowel obstruction, gallstone outside the gallbladder, air in the bile ducts) is sometimes present, however, a nonspecific dilation of the small and large bowel is frequent. For this reason, CT of the abdomen is performed most of the times and 81% (n=46) of cases were diagnosed by CT scan. In some cases, due to the hemodynamic instability, emergent laparotomy was required, for this reason in 7% (n=4) of cases the diagnosis was done during surgery. Other diagnostic modalities were plain abdominal X-ray, endoscopy and gastrografin study, each of these were the main diagnostic methods in only 5% (n=3), 5% (n=3), and 2% (n=1) of cases, respectively (Table 2).

**Table 2:** Main final diagnostic modalities. The main final diagnostic modality reported by each individual study that provided the most accurate diagnosis.

Main diagnostic modality	N (%)
CT abdomen	46 (81)
Laparotomy	4 (7)
Abdominal X-ray	3 (5)
Endoscopy	3 (5)
Gastrografin	1 (2)

The location of the gallstone impaction in the colon was the sigmoid colon, rectosigmoid, or at the descending colon/sigmoid junction in 86% (n=49) of cases, followed by the descending colon (n=4) and transverse colon (n=3) (Table 3). This could be explained by the higher frequency of diverticulosis at the sigmoid segment and consequent increased risk for strictures [10]. The presence of a biliary enteric fistula was described in 40 cases, of which 37 were cholecystocolonic fistulas and three were cholecystoduodenal. The majority of the cholecystocolonic fistulas were formed at the hepatic flexure, including ours. The stone size varied, when using the largest reported diameter, it ranged from 2 to 7 cm, with an average of 4.24 cm. 

**Table 3:** Location of the stone in the colon.

Stone location	N (%)
Sigmoid / descending sigmoid junction / rectosigmoid	49 (86)
Descending colon	4 (7)
Transverse colon	3 (5)

Similar to gallstone ileus occurring at the small intestine, the mainstay of treatment in colonic gallstone ileus is enterolithotomy which consists of a longitudinal colotomy proximal to the point of stone impaction followed by stone removal and closure of the colotomy, however, partial colectomy is sometimes needed and Hartmann’s procedure is common. Owing to advances in endoscopic procedures, the use of colonoscopy for stone removal has been increasing, nevertheless with variable success. Of the 57 cases, 40 (70%) were treated with laparotomy of which four died during the hospitalization, a total of 32 patients had an attempted colonoscopy for treatment but it was only successful in removing the stone in eight patients (14%) and one patient died. Two patients had successful treatment with laparoscopy, one with trephine loop colostomy, five patients had conservative treatment, and one case did not clarify the type of surgery (Table 4). Table 5 summarizes the main characteristics of reported cases of colonic gallstone ileus.

**Table 4:** Final treatment modalities.

Treatment	N (%)
Laparotomy	40 (70)
Endoscopy	8 (12); attempted in 32 (56)
Conservative	5 (9)
Laparoscopy	2 (4)
Trephine loop colostomy	1 (2)

**Table 5:** Main characteristics of the reported cases of colonic gallstone ileus. The main characteristics of the reported cases of colonic gallstone ileus included age, diagnostic modality, stone location and size, treatment, and outcome. When the location of the stone was around the rectosigmoid area it was referred to as sigmoid.

Study	Age	Gender	Diagnosis	Location	Stone size (cm)	Final treatment	Outcome
1	82	Male	CT abdomen	Sigmoid	7	Laparotomy	Discharged
2	90	Female	Gastrografin enema	Sigmoid	3.2	Laparotomy	Discharged
3	87	Female	CT abdomen	Sigmoid	5	Laparotomy	Discharged
4	72	Female	CT abdomen	Sigmoid	2.2	Laparotomy	Discharged
5	85	Female	CT abdomen	Descending and sigmoid	6	Laparoscopic	Discharged
6	54	Female	CT abdomen	Descending and sigmoid	4.5	Endoscopic	Discharged
7	86	Female	CT abdomen	Sigmoid	3.7	Laparotomy	Discharged
8	88	Female	CT abdomen	Sigmoid	4.4	Trephine loop colostomy	Discharged
9	78	Female	CT abdomen	Descending colon	6	Laparotomy	Died
10	80	Female	CT abdomen	Sigmoid	2	Laparotomy	Discharged
11	80	Female	CT abdomen	Sigmoid	3	Laparotomy	Discharged
12	92	Male	CT abdomen	Sigmoid	3.8	Laparotomy	Discharged
13	94	Female	CT abdomen	Sigmoid	6	Endoscopic with lithotripsy	Discharged
14	95	Female	CT abdomen	Sigmoid	3.5	Conservative	Died
15	77	Female	X-Ray abdomen		3	Endoscopy	Unknown
16	92	Male	CT abdomen	Sigmoid	4.1	Endoscopy with lithotripsy	Discharged
17	85	Male	CT abdomen	Sigmoid	4	Conservative	Discharged
18	86	Male	CT abdomen	Sigmoid	3.3	Laparotomy	Discharged
19	88	Female	Laparotomy	Transverse colon	7	Laparotomy	Discharged
20	49	Female	CT abdomen	Sigmoid	4	Laparotomy	Discharged
21	92	Male	CT abdomen	Descending colon	2.5	Laparotomy	Discharged
22	73	Male	CT abdomen	Sigmoid	4	Endoscopy with lithotripsy	Discharged
23	83	Female	CT abdomen	Sigmoid	Unknown	Endoscopy laser lithotripsy	Died
24	81	Female	CT abdomen	Sigmoid	2.5	Laparotomy	Discharged
25	91	Female	CT abdomen	Sigmoid	4.6	Laparotomy	Discharged
26	86	Female	CT abdomen	Sigmoid	3.7	Laparotomy	Discharged
27	86	Female	CT abdomen	Sigmoid	3	Laparotomy	Discharged
28	76	Female	CT abdomen	Sigmoid	3.5	Laparotomy	Discharged
29	70	Female	CT abdomen	Descending colon	3	Laparotomy	Died
30	85	Female	Laparotomy	Sigmoid	6.5	Laparotomy	Discharged
31	80	Female	Laparotomy	Sigmoid	2.3	Laparotomy	Discharged
32	78	Female	X-Ray abdomen	Sigmoid	4	Laparotomy	Unknown
33	87	Female	CT abdomen	Sigmoid	4.2	Laparotomy	Unknown
34	65	Female	Colonoscopy	Descending colon	Unknown	Conservative	Discharged
35	65	Male	CT abdomen	Sigmoid	7	Laparotomy	Discharged
36	80	Female	CT abdomen	Sigmoid	5	Endoscopy with extracorporeal lithotripsy	Discharged
37	88	Female	CT abdomen	Sigmoid	4.7	Laparotomy	Died
38	68	Female	CT abdomen	Sigmoid	6	Laparoscopic	Discharged
39	80	Female	CT abdomen	Sigmoid	2	Laparotomy	Discharged
40	72	Male	CT abdomen	Sigmoid	4	Laparotomy	Unknown
41	73	Male	CT abdomen	Transverse Colon	7	Laparotomy	Discharged
42	89	Female	CT abdomen	Sigmoid	5	Laparotomy	Discharged
43	77	Female	Sigmoidoscopy	Sigmoid	3.8	Laparotomy	Unknown
44	77	Female	CT abdomen	Sigmoid	2.8	Laparotomy	Discharged
45	69	Female	CT abdomen	Sigmoid	3.6	Laparotomy	Discharged
46	89	Female	CT abdomen	Sigmoid	5	Laparotomy	Discharged
47	69	Female	CT abdomen	Sigmoid	4.8	Manual Removal	Discharged
48	67	Male	CT abdomen	Sigmoid	7	Sigmoidoscopy	Unknown
49	78	Male	CT abdomen	Sigmoid	3	Laparotomy	Unknown
50	75	Female	CT abdomen	Sigmoid	Unknown	Laparotomy	Unknown
51	80	Unknown	CT abdomen	Sigmoid	Unknown	Surgery	Unknown
52	87	Male	Laparotomy	Sigmoid	Unknown	Laparotomy	Unknown
53	92	Female	CT abdomen	Transverse colon	Unknown	Conservative	Discharged
54	78	Male	X-ray abdomen	Sigmoid	Unknown	Laparotomy	Discharged
55	87	Female	CT abdomen	Sigmoid	Unknown	Laparotomy	Discharged
56	88	Female	Laparotomy	Sigmoid	Unknown	Lapartotomy	Discharged
57 (Ours)	89	Male	CT abdomen	Sigmoid	3.4	Laparotomy	Died

Sigmon et al. used an adult diagnostic gastroscope and performed several attempts to capture the stone with a Roth Net which were unsuccessful, they then used an adult therapeutic gastroscope with a Talon grasping device that was again, unsuccessful. With the last gastroscope and a hexagonal AcuSnare they were able to snare the stone and remove it entirely in one piece [2]. Balzarini et al. used a different approach during their endoscopic procedure, an Olympus videoscope and CO2 insufflation were utilized, and a 3 x 6 French lithotripsy extraction basket was inserted through the biopsy channel of the videoscope, the stone was caught with the basket, and lithotripsy was successfully performed [11]. Zielinski et al. successfully removed a gallstone from the sigmoid via intracolonic electrohydraulic lithotripsy provided by a French electrohydraulic lithotripsy probe that was also inserted in an Olympus gastroscope [12]. In our case, the several attempts to capture the stone were unsuccessful which could be explained by the degree of stenosis at the site of impaction, furthermore, the mucosa had increased friability and was at risk for perforation.

The overall mortality related to gallstone ileus had been decreasing, In the nineteenth century, gallstone ileus had a high mortality rate of around 44% [3]. In the large review of 1001 cases by Reisner et al. in 1994, the reported mortality rate had decreased to 18% [1]. Moreover, the very large study of Halabi et al. showed a mortality rate of 6.67% [6]. In this review, 48 cases reported the patient’s outcome, from these six died during the hospitalization which corresponds to a mortality rate of 12.5%. 

## Conclusions

Management of colonic gallstone ileus in high-risk patients is challenging, particularly due to the common advanced age and several comorbidities that many of these patients possess which make them poor surgical candidates. Nonetheless, given the low success rate with other therapeutic modalities, laparotomy remains the standard approach which may involve enterolithotomy or even partial colectomy if the intestinal wall is perforated or necrotic. Endoscopic treatment has been reported in the literature with only a few successful cases mainly using mechanical or electrohydraulic lithotripsy. Several factors influence this outcome including stone size, presence of colonic stricture, time for treatment, and lack of expertise or availability. In our patient, the failure of the endoscopic approach was likely a combination of these factors. An endoscopic approach using the available tools and expertise should be attempted in the presence of surgical support due to the risk of intestinal perforation and often failed endoscopic attempts. Conservative management is not recommended as the spontaneous evacuation of the stone is rare and treatment delays will most likely lead to severe complications. 
